# Study on Mechanism of Mechanical Property Enhancement of Expansive Soil by Alkali-Activated Slag

**DOI:** 10.3390/ma18040800

**Published:** 2025-02-12

**Authors:** Quan Shen, Chaohui Wang, Yuan Yan, Shiyuan Yi, Ke Teng, Chaojun Li

**Affiliations:** 1 School of Civil Engineering, Hunan University of Technology, Zhuzhou 412007, China; m22085900005@stu.hut.edu.cn (C.W.); m24081400014@stu.hut.edu.cn (S.Y.); abaddontank@126.com (K.T.); 2221980@chester.ac.uk (C.L.); 2 School of Civil Engineering, Central South University, Changsha 410083, China; 3 Collaborative Innovation Center, Hunan Automotive Engineering Vocational College, Zhuzhou 412001, China

**Keywords:** expansive soil, alkaline activator, slag, UCS, enhancement mechanism, C-S-H gel, C-A-S-H gel, microstructure, modulus, engineering improvement

## Abstract

Expansive soil poses significant challenges for engineering due to its susceptibility to swelling and shrinkage. This study aims to explore effective methods for improving its mechanical properties using single alkaline activators, single slag, and their combination. Laboratory experiments were conducted to evaluate the unconfined compressive strength (UCS) and analyze curing mechanisms through X-ray diffraction (XRD) and scanning electron microscopy (SEM). The results demonstrate that all three treatments enhance soil strength, with the combination of alkali-activated slag being the most effective, followed by the single alkaline activator and single slag. Optimal dosages were determined as 15% for the activator and slag individually and 15% activator combined with 20% slag, yielding the densest structure and highest UCS. The activator’s modulus of 1.5 was found to be optimal, and strength improved further with extended curing time. A microscopic analysis revealed that alkaline activation formed gel-like substances and dense needle-like structures, while slag generated CaCO_3_ and Ca(OH)_2_. The combination produces a synergistic effect, creating substantial amounts of C-S-H, C-A-S-H gel, and dense needle-like structures, which enhance soil compactness and strength by binding particles and filling voids. These findings provide insights into the curing mechanisms and offer practical solutions for improving expansive soil in engineering applications.

## 1. Introduction

Expansive soil is the most widely distributed problematic soil in the world, found in regions such as the United States, India, South Africa, and Australia [1,2]. Expansive soil is rich in montmorillonite and montmorillonite groups, which have high moisture retention and weak interface bonding properties [3]. Due to construction on expansive soil, the estimated annual losses in the United States are USD 9 billion, USD 1 billion in China, and USD 500 million in the United Kingdom. From 1970 to 2000, the total annual losses increased by approximately 140%, with USD 4 billion in losses attributed to road structures in the United States alone [4,5]. Research into expansive soil improvement technologies is of great significance in the field of engineering construction. To date, scholars both domestically and internationally have proposed various methods for improving expansive soil based on its material composition, structural characteristics, and expansion–shrinkage mechanism. These methods include physical stabilization, the use of bio-fibers, chemical treatment, and the use of industrial waste materials.

Physical improvement methods, such as the combined use of construction waste and glass fibers, can improve the CBR of expansive soil to 5.0 and reduce its expansion–shrinkage characteristics, making it suitable for first-class highway subgrade and roadbed requirements [6]. The composite improvement of expansive soil with fly ash and sisal fiber can increase its UCS to 357.9 kPa [7]. According to Zhou [8], expansive soil improved with construction waste shows better strength, expansion–shrinkage characteristics, and permeability. However, the improvement effect of fiber addition is difficult to guarantee in terms of uniform distribution, and the improvement is random. The effectiveness of materials like construction waste and fly ash in improving the mechanical properties of expansive soil is limited. Chemical improvement generally uses traditional materials like lime and cement. Zhang [9] found that 6% lime can increase the UCS of expansive soil to 3.98 MPa, but the verification, digestion, and storage treatment of lime during the preparation stage are cumbersome. Chai et al. [10] found that cement has a better solidification effect on expansive soil than lime, followed by sand and gravel. Bi [11] believes that the improvement effect of single lime or cement is much less than the dual incorporation of lime and cement. Zhao [12] used cement, lime, and fly ash to solidify expansive soil and found that the improvement effect of cement and lime + cement was better than that of fly ash. However, the production of lime and cement is associated with a large amount of CO_2_ emissions. Producing one ton of cement and one ton of lime emits 0.95 and 0.79 tons of CO_2_, respectively, and consumes 5000 megajoules and 3200 megajoules of energy [13,14,15,16]. Furthermore, cement production accelerates the depletion in natural resources, with the production of one ton of cement consuming approximately 1.5 tons of limestone and clay [17].

Therefore, finding efficient, low-carbon, environmentally friendly, and low-cost construction methods for solidifying expansive soil using waste resources is currently a research hotspot. In recent years, the application of industrial waste materials such as steel slag powder, fly ash, and slag in the improvement of expansive soil has attracted widespread attention. Studies have shown that steel slag powder can significantly improve the compressive strength of expansive soil, reducing its expansion rate and water sensitivity [18,19]. Additionally, steel slag powder can improve the crack development of expansive soil under dry–wet cycles and enhance its strength [20]. Fly ash has also been found to effectively suppress the expansion characteristics of expansive soil and reduce its plasticity index and expansion rate [21]. However, the effect of solely using these industrial waste materials to solidify expansive soil is often limited. As a result, scholars have proposed accelerating the hydration process through chemical activation [22], further improving the performance of expansive soil. Alkali activators, as an effective chemical activation method, have been widely applied in improving expansive soil. Research shows that steel slag cement activated by NaOH can significantly improve the compressive strength of expansive soil under multiple dry–wet cycles and improve its resistance to strength degradation [23]. Similarly, NaOH-activated fly ash can quickly increase the shear strength of expansive soil, overcoming the slow reaction activity of fly ash [24]. Liu et al. [25] found that the Na_2_SiO_3_ activation of slag–fly ash improved expansive soil strength by 107% compared to slag–fly ash alone. Among different alkali activators, Na_2_SiO_3_ is considered to have the best solidification effect [26]. While studying the mechanical properties of alkali-activated industrial waste-improved expansive soil, scholars have also delved into the enhancement mechanisms. The core mechanism of alkali-activated industrial waste improvement in soil is through alkali materials activating the SiO_2_ and Al_2_O_3_ in industrial waste; disrupting their tetrahedral structures; breaking Si-O-Si bonds, Si-O-Al bonds, and Al-O-Al bonds; depolymerizing them into plasma-state monomers that easily react with Ca^2+^; and subsequently forming gel bodies through polycondensation that strengthen the soil, thus improving its strength. Fernández et al. [27] proposed through scanning electron microscopy (SEM) and transmission electron microscopy (TEM) testing that the reaction process of alkali-activated fly ash consists of three stages: dissolution, diffusion, and precipitation. Further studies show that chemical activation can alter the glass structure of fly ash, activating its reactivity and improving issues such as long hardening reaction periods and poor durability [28]. X-ray diffraction (XRD) and scanning electron microscopy (SEM) analyses by Wang et al. [29] revealed that the strength improvement of expansive soil after the NaOH activation of fly ash is due to the formation of C-S-H and (C,N)-A-S-H gels, which enhanced the internal structure of the soil and reduced porosity. Chai et al. [30] showed that after activating fly ash and steel slag with NaOH, new materials like calcium carbonate whiskers, needle-like calcium silicate hydrate, and amorphous silicate-aluminate polymers were generated, improving the density of the soil and enhancing its mechanical properties. Mazhar et al. [31] studied the use of alkali-activated steel slag–fly ash-solidified expansive soil as roadbed material, measuring its strength characteristics and conducting load tests. Their research indicated that the improvement mechanism was the generation of C-S-H gels, calcium carbonate whiskers, and sodium zeolite filling gaps, which strengthened the material and formed a dense structure, thus increasing the strength of the soil. Du [32] revealed that the curing of expansive soil with lime-activated metakaolin–steel slag composites improved soil strength by generating C-S-H, C-A-S-H, Aft, and other hydration products, which enhanced the cementation effect between soil particles. Xiang et al. [33] found that lime activated the cementitious materials in steel slag, forming C-S-H colloids that strengthened the coagulation effect between particles, improving the performance of steel slag–lime composites in improving expansive soil. Zhang et al. [34] proposed that the improvement mechanism of lime-activated blast furnace slag-solidified expansive soil was also through hydration reactions forming C-S-H gels, which solidified and cemented dispersed soil particles into a network structure, effectively improving soil strength. Moreover, Mazhar et al. [35] added fibers to alkali-activated slag–fly ash for cooperative curing of expansive soil, finding that new molecular bonds were formed in the chemically treated fibers, overcoming early biodegradation issues and improving the interlocking density and durability between soil and fibers. The improved soil generated new elemental and crystalline phases, forming a network of channel bridges that enhanced the soil’s resistance to compressive, shear, and tensile loads. Hamed et al. [36] studied NaOH-activated fly ash–slag-solidified cohesive soil, showing that the generated C-S-H and N-A-S-H gels filled soil pores, creating a denser structure and texture, improving compressive strength. Miraki et al. [37] discovered that the mechanism of NaOH-activated volcanic ash–slag-solidified soil involved generating N-A-S-H and C-(A)-S-H gels, where N-A-S-H gels provided the network structure and C-(A)-S-H gels enhanced soil strength and rigidity. The interaction between these gels ensured a dense microstructure in the soil, thus improving its overall performance.

Expansive soils contain a significant amount of SiO_2_ and Al_2_O_3_, which may react under the influence of alkaline activators, leading to solidification. Therefore, this study explores the direct solidification of expansive soil using alkaline activators and investigates the solidification mechanism in detail. The solidification effect of alkaline-activated expansive soil is compared with that of slag and alkaline-activated slag-based geopolymers, aiming to optimize its performance. The study evaluates the effectiveness of the three solidification methods through unconfined compressive strength (UCS) tests and examines the effects of the modulus, dosage, and curing time on solidification, in order to determine the optimal mix ratio. Additionally, the solidification mechanism is analyzed using X-ray diffraction (XRD) and scanning electron microscopy (SEM). The findings will enhance the understanding of expansive soil solidification techniques and provide theoretical support for the activation mechanisms of industrial waste, promoting the resource utilization and harmless treatment of slag and expansive soils.

## 2. Material and Methods

### 2.1. Materials

**Figure 1:**
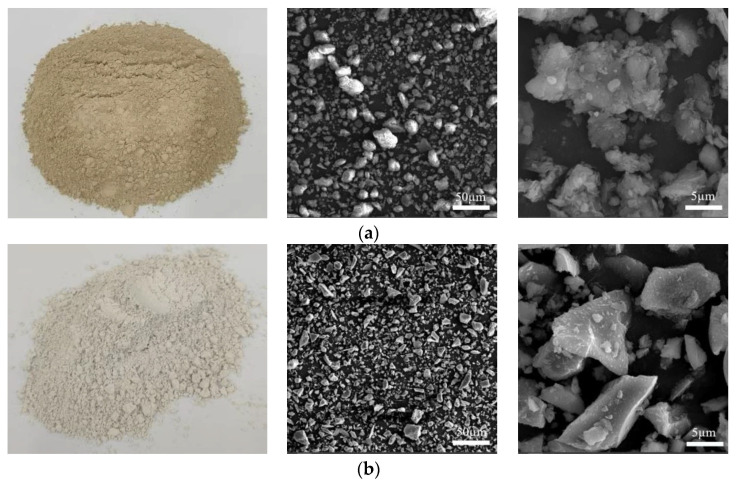
Images of the SEM analysis of expansive soils and GGBS (1000× and 10,000×): (**a**) expansive soil and SEM (1000× and 10,000×); (**b**) GGBS and SEM (1000× and 10,000×).

The expansive soil used in this study was collected from the Baise region of Guangxi, characterized by a flaky structure. The basic physical and mechanical parameters of this soil were determined according to standard geotechnical testing methods and are summarized in Table 1. According to the Unified Soil Classification System (USCS), the soil was classified as expansive silt (ML). The slag (GGBS) was derived from ground granulated blast furnace slag (Henan Wuhu Environmental Protection Technology Co., Ltd., Zhengzhou, China), a byproduct of iron smelting. GGBS has a dense, glassy structure. The alkali activator (L) was prepared by mixing Na_2_SiO_3_ (Henan Borun New Materials Co., Ltd., Zhengzhou, China) powder with analytical reagent-grade NaOH (Shandong Binhua Chemical Co., Ltd., Binzhou, China) in solid pellet form. Figure 1 shows the expansive soil, GGBS, and their microstructures (SEM magnifications of 1000× and 10,000×).

Expansive soil and GGBS both contain high levels of SiO_2_ and Al_2_O_3_, making them suitable for activation with alkaline activators (L). The activation effect improves as the particle size of the expansive soil decreases; therefore, the expansive soil was ground to a near-powder consistency for the experiments. Additionally, GGBS has a significant content of CaO, allowing it to also function as a curing agent for expansive soil. The primary chemical components of expansive soil and GGBS are summarized in Table 2.

### 2.2. Experimental Design

This study shows the effects of three curing methods—a single incorporation of an alkaline activator (L), single incorporation of slag (GGBS), and alkaline-activated slag-based geopolymers (LGGBS)—on the unconfined compressive strength (UCS, Shenzhen Wance Testing Machine Co., Ltd., Shezhen, China) of expansive soil. The enhancements in UCS will be analyzed using scanning electron microscopy (SEM, TESCAN, Shanghai, China) and X-ray diffraction (XRD, Rigaku Corporation, Tokyo, Japan) techniques to understand the underlying micro-mechanisms. The modulus of the alkaline activator, defined as the ratio of SiO_2_ to Na_2_O, serves as a crucial parameter for assessing the alkalinity of the activator. For the L and LGGBS curing experiments, we first examine the influence of different moduli on the UCS of expansive soil with a fixed L content of 15%. This helps identify the optimal modulus, which will be used in subsequent experiments as a constant for the L solution, while also exploring the effects of varying L dosages on UCS. In the case of GGBS stabilization, the slag is added in predetermined proportions. The moduli for the L solution are set at 1.0, 1.2, 1.5, 1.7, and 2.0, with dosages of 5%, 10%, 15%, and 20%. The GGBS content will be varied at 10%, 15%, and 20%. The curing durations are established at 7 days, 14 days, and 28 days. The detailed experimental design is presented in Table 3.

### 2.3. Sample Preparation and Curing

In this study, the expansive soil was first dried in an oven at 105 °C and then mixed with deionized water to prepare L solutions of varying moduli and content, allowing them to stand for 24 h to release hydration heat [38,39]. For GGBS-stabilized expansive soil, the dried soil samples were uniformly blended with GGBS in the appropriate proportions. For L-stabilized expansive soil, the prepared L solution was evenly sprayed onto the dried soil samples. In the case of LGGBS stabilization, the dried soil was first mixed with GGBS and then the L solution was sprayed evenly. All three types of stabilized soils were treated with solutions corresponding to a natural moisture content of 33.4%, ensuring thorough mixing before sealing the mixture for 24 h. The amount of L was calculated based on the dry weight of the soil. The mixture was compacted in four layers using a triaxial compactor, targeting a natural dry density of 1.41 g/cm^3^, with sample dimensions of D = 39.1 mm and H = 80 mm. After preparation, the samples were sealed with a thin film and cured for 2 days, after which the film was removed and the samples were placed in sealed transparent plastic containers for continued curing at room temperature. Three parallel samples were prepared for each group, and the average values were calculated within acceptable error limits; if discrepancies were significant, the samples were re-prepared. Strength testing was conducted at curing durations of 7, 14, and 28 days. For the samples cured for 28 days, fragments were ground into powder for an XRD phase analysis, while selected fragments, measuring less than 1 cm in length, width, and height with smooth surfaces, were used for SEM testing.

## 3. Results and Analysis

### 3.1. Volume Stability Test

**Figure 2:**
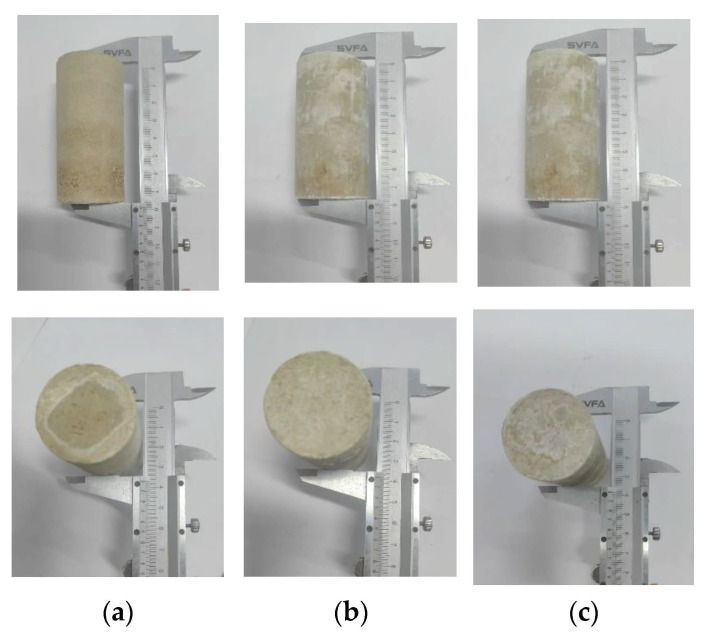
Volume change conditions: (**a**) alkali-activated soil; (**b**) slag-stabilized soil; (**c**) alkali-activated slag-based geopolymer-stabilized soil.

In research, we conducted volume change tests on both natural expansive soil samples and expansive soil samples treated with different solidification methods to assess the effects of alkali activators, slag, and alkali-activated slag-based geopolymers on the volume stability of expansive soil, as shown in Figure 2.

After 28 days of curing, the natural expansive soil samples exhibited a volume shrinkage rate of 20.53%. Upon immersion in water, these samples rapidly disintegrated, displaying significant characteristics of swelling when wet and shrinking when dry. This indicates that, in its natural state, expansive soil is highly susceptible to volume deformation, especially under changing water conditions. In contrast, the samples treated with solidification methods showed remarkable volume stability. As shown in Figure 2, the L-stabilized soil, GGBS-stabilized soil, and LGGBS-stabilized soil samples exhibited no significant volume changes after 28 days of curing, nor did they show any noticeable volume changes when immersed in water. This suggests that the swelling and shrinking characteristics of expansive soil have been significantly improved through solidification treatment, resulting in enhanced volume stability. In particular, the LGGBS-stabilized soil demonstrated more pronounced solidification effects. The soil maintained a stable volume after solidification, effectively suppressing the volume deformation of expansive soil.

### 3.2. UCS Testing

#### 3.2.1. Effects of Alkaline Activator on UCS of Expansive Soil

**Figure 3:**
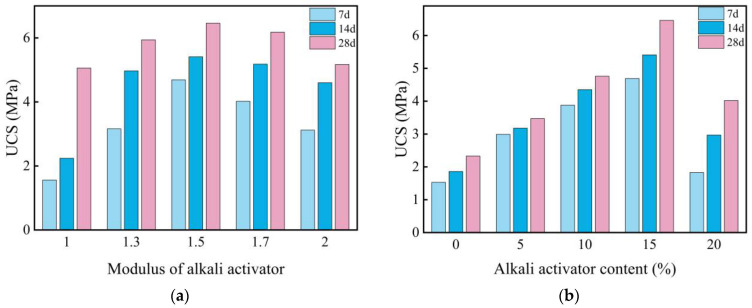
Influence of different modulus and content of alkaline activators on UCS of stabilized expansive soil: (**a**) influence of modulus on UCS of stabilized expansive soil; (**b**) influence of alkaline activator content on UCS of stabilized expansive soil.

Figure 3a illustrates the UCS of the L-stabilized soil with different moduli after curing for 7, 14, and 28 days. It is evident that, across various curing durations, the UCS of the stabilized soil initially increases and then decreases with an increasing modulus. The maximum UCS of 6.46 MPa was achieved at a modulus of 1.5, representing a 177% increase compared to pure expansive soil. However, when the modulus exceeds 1.5, the UCS declines, albeit remaining higher than that of pure expansive soil. This indicates that higher alkalinity does not necessarily lead to better activation; rather, an optimal alkaline environment is essential to maximize the reactivity of active components such as SiO_2_ and Al_2_O_3_, leading to the formation of gel-like substances that enhance soil strength. Furthermore, as the curing period increases, the UCS of the L-stabilized soil gradually improves, while the influence of the modulus on UCS diminishes. During the 7-day curing period, the UCS exhibits significant variation with changes in the modulus, showing linear growth. In contrast, the trends become more subdued at 14 and 28 days, with reduced differences in UCS across moduli and decreased sensitivity to modulus changes.

Thus, the optimal modulus was determined to be 1.5, and different content of L solutions were prepared based on this modulus. Figure 3b presents the UCS of the stabilized soils with varying L content after curing for 7, 14, and 28 days. Compared to pure expansive soil, the UCS of the L-stabilized soil demonstrates varying degrees of improvement, indicating that L effectively activates the SiO_2_ and Al_2_O_3_ in expansive soil to form gel-like materials that enhance strength. As the L content increases, the UCS initially rises and then declines, displaying a linear trend, peaking at a 15% L content. However, at a content of 20%, a significant drop in UCS is observed. The main reason is that the high viscosity of the L hinders the formation of a homogeneous silica gel matrix, which leads to a reduction in strength gain [40]. This further confirms that an appropriate alkaline environment is necessary to optimize the activation of SiO_2_ and Al_2_O_3_ in expansive soil, and excessive L does not yield favorable results. Similarly, the UCS of the stabilized soil increases with longer curing periods, though the growth rate is relatively small.

#### 3.2.2. Effects of Slag on the UCS of Expansive Soil

**Figure 4:**
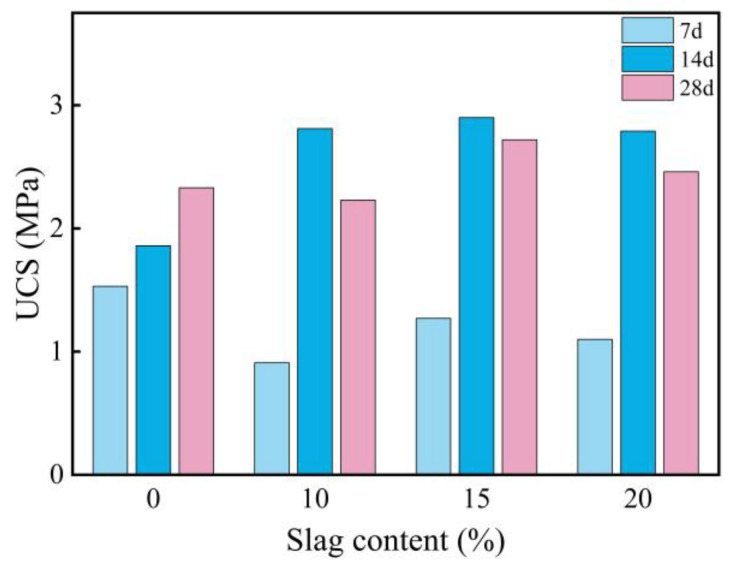
Influence of slag content on the UCS of stabilized expansive soil.

Figure 4 illustrates the UCS of GGBS-stabilized soil after curing for 7, 14, and 28 days. The results indicate that the addition of GGBS improves the UCS of expansive soil, although the increase is modest. As the GGBS content increases, the UCS initially rises and then declines, with the optimal performance observed at a 15% GGBS content. Notably, the UCS of GGBS-stabilized soil shows a similar pattern of an increase followed by a decrease across different curing durations. The UCS reaches its peak at 14 days but declines by 28 days, suggesting a potential deterioration of the GGBS-stabilized soil during long-term curing. Additionally, after 7 days of curing, the UCS of all three GGBS content variations remains lower than that of pure expansive soil. At 28 days, only the UCS of the 10% GGBS content remains below that of pure expansive soil, while the UCS of the 14-day cured GGBS-stabilized soil exceeds that of the pure expansive soil. These findings indicate that using GGBS alone for stabilizing expansive soil may not yield satisfactory results, as the reactivity of the GGBS does not reach its full potential. To enhance its activity, the use of an activator is necessary.

#### 3.2.3. Effect of Alkali-Activated Slag-Based Geopolymer on UCS of Expansive Soil

**Figure 5:**
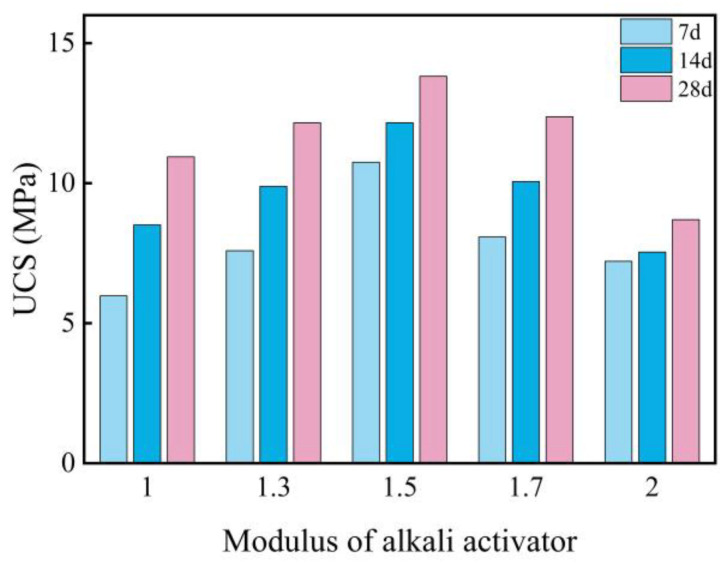
Influence of modulus on UCS of stabilized expansive soil.

Figure 5 presents the UCS of LGGBS-stabilized soil with varying moduli after curing for 7, 14, and 28 days. The results indicate that, under the conditions of 15% L and 20% GGBS, the UCS of the stabilized soil exhibits a trend of initially increasing and then decreasing as the modulus changes. As the modulus increases from 1.0 to 1.5, the UCS shows linear growth. However, when the modulus exceeds 1.5, the UCS begins to decline. This suggests that a moderate increase in alkalinity facilitates the activation of GGBS, thereby enhancing soil strength. Conversely, excessively high alkalinity may lead to an over-reaction, resulting in the formation of unstable gel structures that weaken soil strength. Across all modulus ranges, the UCS of LGGBS-stabilized soil is significantly higher than that of pure expansive soil and L-stabilized soil, demonstrating the effective synergistic effect of LGGBS in enhancing UCS. Furthermore, the influence of different curing durations on UCS exhibits a consistent pattern, with the maximum UCS achieved at a modulus of 1.5 for all curing times. This result further validates the optimal role of the L solution with a modulus of 1.5 in promoting slag reactivity, optimizing microstructure, and improving macroscopic mechanical properties. Therefore, the selection of an appropriate modulus is critical for optimizing the UCS performance of LGGBS-stabilized expansive soil. Additionally, the UCS of LGGBS-stabilized soil shows linear growth with extended curing periods.

**Figure 6:**
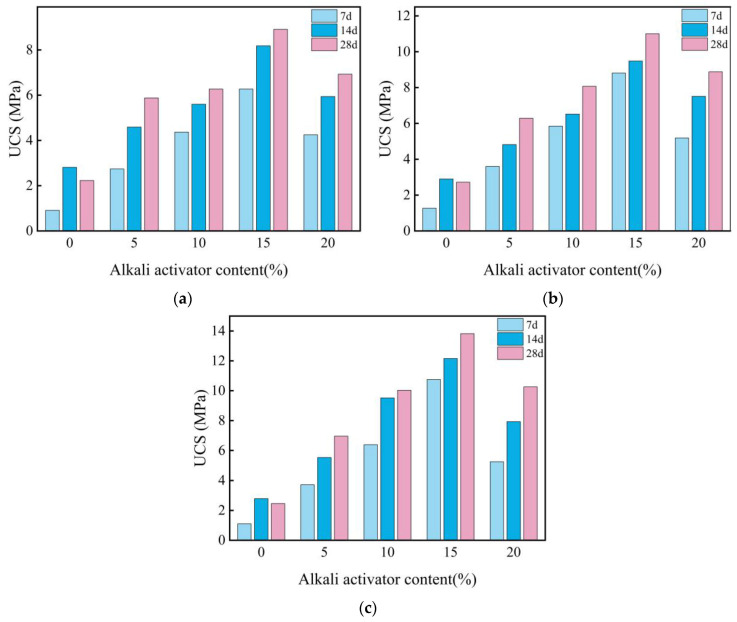
Influence of alkali activator content on UCS of stabilized expansive soil with varying slag contents: (**a**) 10% GGBS; (**b**) 15% GGBS; (**c**) 20% GGBS.

Based on these findings, the modulus is fixed at 1.5 for subsequent experiments involving different dosages of the L solution. Figure 6 illustrates the UCS of stabilized soil under varying L and GGBS contents and curing durations. The results reveal that the UCS of LGGBS-stabilized soil initially increases and then decreases with increasing L content, with the optimal performance observed at 15%. Adequate L effectively activates GGBS reactivity, leading to the formation of gel-like substances that enhance soil strength. In contrast, excessive L may result in an over-reaction and the generation of excessive byproducts, ultimately weakening soil strength. Regarding GGBS content, the UCS consistently increases with higher GGBS proportions across all L dosages, indicating that the addition of GGBS provides more active components, significantly enhancing soil strength. Additionally, with longer curing periods, the UCS exhibits linear growth, further confirming that prolonged curing facilitates the ongoing alkaline activation reaction, thereby enhancing soil strength.

Overall, LGGBS significantly improved the UCS of expansive soil. The appropriate selection of L and GGBS dosages, along with suitable curing durations, is crucial for improving UCS. These findings provide important experimental evidence for further optimizing soil stabilization techniques for expansive soils.

### 3.3. Analysis of Enhancement Mechanisms

#### 3.3.1. XRD Analysis

**Figure 7:**
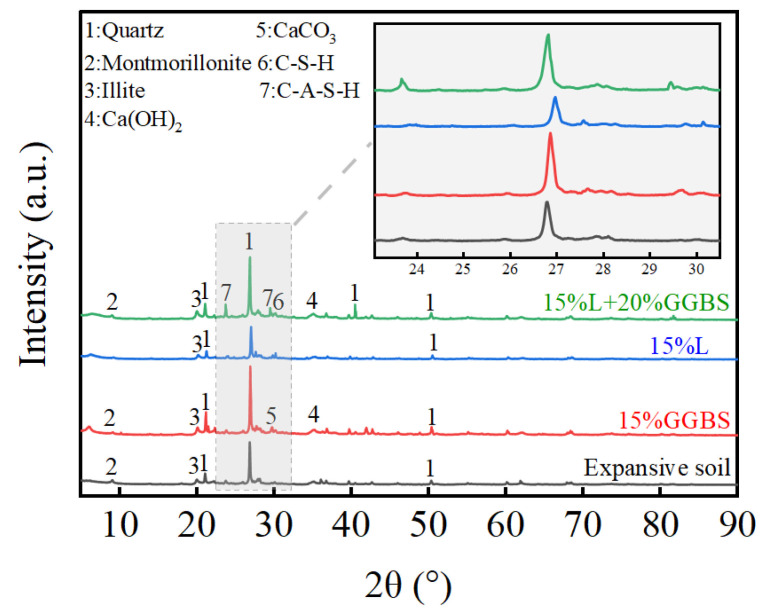
XRD patterns.

To further investigate the mechanisms by which L, GGBS, and LGGBS enhance the strength of expansive soil, an X-ray diffraction (XRD) analysis was conducted on samples cured for 28 days. The XRD patterns are shown in Figure 7. An analysis of the diffraction patterns reveals the formation of calcium silicate hydrate (C-S-H), calcium aluminate silicate hydrate (C-A-S-H), calcium hydroxide (Ca(OH)_2_), and calcium carbonate (CaCO_3_) in the stabilized soil. The formation of these new mineral phases occurs through the following processes: (i) the dissolution of SiO_2_ and Al_2_O_3_ in a highly alkaline environment; (ii) the reaction of CaO in the slag with silicate and aluminate monomers to form reaction products; (iii) some CaO reacting with silicate monomers to generate C-S-H; (iv) Na⁺ ions balancing the charge and being absorbed by the silico-aluminate phases to form N-A-S-H; and (v) some Ca^2^⁺ ions potentially replacing Na⁺ to form calcium-aluminosilicate hydrate (C-A-S-H) [41,42,43]. The results indicate that the expansive soil primarily contains minerals such as quartz, montmorillonite, and illite, which remain present after stabilization. The GGBS-stabilized soil shows the presence of CaCO_3_ and Ca(OH)_2_. The L-stabilized soil without slag does not show the formation of new compounds. However, the XRD pattern of the LGGBS-stabilized soil shows both C-A-S-H and C-S-H gel [44], along with Ca(OH)_2_ in the same diffraction range. These results suggest that the silico-aluminate glass in the slag dissolves in the alkaline environment, leading to the breaking of Si-O and Al-O bonds within the glass structure. This process activates the potential reactivity, leading to the re-polymerization of hydration products such as C-A-S-H and C-S-H gels. These gel-like substances fill the pores of the soil and enhance the bonding between particles, significantly improving UCS of the soil. Overall, LBBS leads to a substantial enhancement of the mechanical properties of expansive soil, providing strong mineralogical support for the effectiveness of alkaline activation stabilization techniques.

#### 3.3.2. Qualitative Analysis via SEM

**Figure 8:**
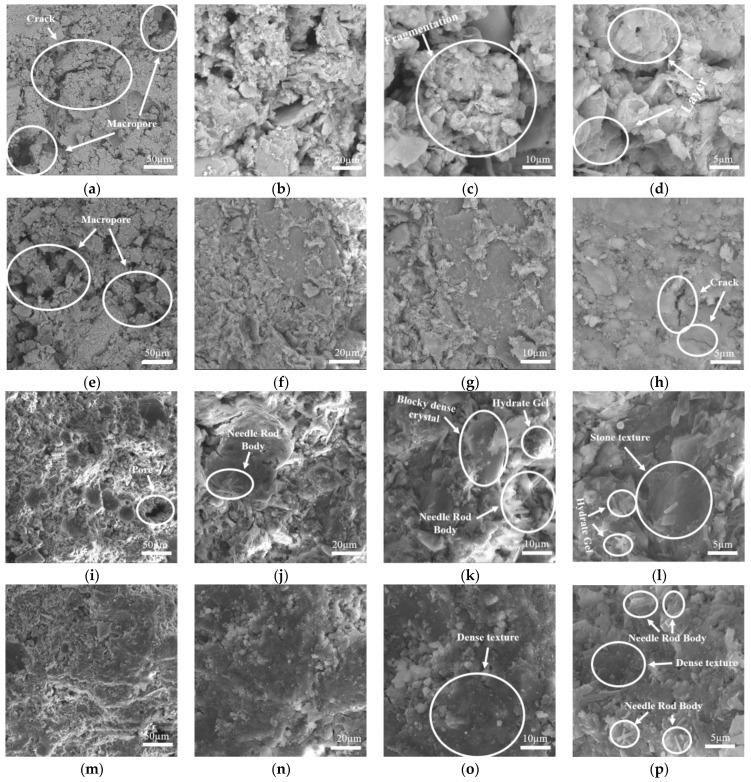
SEM analysis of different types of stabilized expansive soil: (**a**–**d**) expansive soil (1000×, 3000×, 5000×, 10,000×); (**e**–**h**) GGBS-stabilized soil (1000×, 3000×, 5000×, 10,000×); (**i**–**l**) L-stabilized soil (1000×, 3000×, 5000×, 10,000×); (**m**–**p**) LGGBS-stabilized soil (1000×, 3000×, 5000×, 10,000×).

Figure 8 shows the results of a comparative analysis conducted using scanning electron microscopy (SEM) on representative soil samples. The analysis included pure expansive soil and samples stabilized using three different methods, all cured for 28 days.

From Figure 8a–d, it is evident that the microstructure of pure expansive soil contains numerous flaky montmorillonite crystals. The soil particles are primarily composed of fine clay, exhibiting a rough surface and a loose skeletal structure characterized by a significant number of pores and deep cracks. The connections between particles are not tight, primarily arranged in face-to-face or edge-to-edge layered stacking. These flaky structures are connected merely by smaller flakes, with clear patterns at the junctions, displaying distinct signs of looseness and fragmentation. In contrast, Figure 8e–h show that the connections between soil particles in GGBS-stabilized soil are tighter than in pure expansive soil, forming continuous large flaky structures with a small amount of flocculent cementing material. These cementing materials link some flaky structures to the soil particles, reducing the presence of large pores. However, despite these structural improvements, numerous small pores and some minor cracks remain, indicating that the overall structure is still relatively loose. Figure 8i–l illustrate that under the influence of L, the soil structure undergoes significant changes. Large pores nearly disappear, leaving only a few small pores. Although some cracks persist, they are limited to the surface and are quite shallow. Most areas of the soil exhibit a tightly connected state, accompanied by a considerable presence of dense block-like structures, with some regions displaying a stone-like texture. Additionally, a large number of needle-like structures are embedded within the soil, while a small amount of gel-like products are formed. The gel products enhance the bond between particles, and together with the hard needle-like and rod-like substances, they strengthen the soil structure, significantly improving its compressive strength. Figure 8m–p reveal even more pronounced improvements in the microstructure of LGGBS-stabilized soil. While a few small pores remain, the soil particles generally appear to be interconnected, exhibiting greater density as the magnification increases. The particles are closely packed together, with only a few flaky montmorillonite structures visible. Needle-like structures are still present within the soil, alongside some dense crystalline materials. These observations further validate the synergistic enhancement of soil structure provided by an alkaline-activated slag-based geopolymer.

In summary, through the analysis of chemical composition and microstructure, the mechanisms by which the three stabilization methods enhance the UCS of expansive soil are revealed. The mechanical properties of GGBS-stabilized soil are improved by the presence of CaCO_3_ and Ca(OH)_2_, while L-stabilized soil is strengthened by hard needle-like and rod-like structures as well as gel-like substances. LGGBS-stabilized soil benefits from the combined effects of C-A-S-H gel, C-S-H gel, and Ca(OH)_2_. This confirms that the order of stabilization effectiveness is LGGBS > L > GGBS for expansive soil, and further validates the superior advantages of the alkaline-activated slag-based geopolymer at the microstructural level.

## 4. Conclusions

This research focuses on stabilizing expansive soil with alkaline activators, slag, and an alkaline-activated slag-based geopolymer. The conclusions, derived from UCS, XRD, and SEM test results, are presented below:

(a) The three stabilizing agents—L, GGBS, and LGGBS—can effectively suppress the swelling and shrinking behavior of expansive soil, thereby maintaining volume stability.
(b) All three stabilizing agents—L, GGBS, and LGGBS—effectively enhance the mechanical properties of expansive soil. The presence of alkaline activators significantly promotes the dissociation and polymerization reactions of slag, with the order of effectiveness being LGGBS > L > GGBS.
(c) An appropriate alkaline environment facilitates the breakage of Si-O and Al-O bonds in GGBS. Adequate amounts of L and GGBS can react sufficiently to form amorphous aluminosilicate polymer gels that bind soil particles, thereby improving the soil’s strength. The optimal modulus for L is determined to be 1.5, with the best individual dosages being 15% for both L and GGBS, and a combination dosage of 15% L and 20% GGBS. The UCS of L-stabilized soil and LGGBS-stabilized soil increases with curing time, while the effect of the alkali activator modulus on UCS diminishes over time.
(d) For expansive soil stabilized with only GGBS, the optimal dosage is 15%, though a degradation phenomenon occurs with extended curing time, as indicated by higher UCS values at 14 days compared to 28 days. However, the addition of L leads to a linear increase in UCS with increased GGBS content, with no observed degradation.
(e) XRD and SEM analyses indicate that L-stabilized soil improves the UCS of expansive soil through needle-like structures and gel-like substances, while GGBS-stabilized soil enhances its UCS through CaCO_3_ and Ca(OH)_2_. LGGBS-stabilized soil, on the other hand, increases its UCS through products such as amorphous aluminosilicate polymer gels (C-S-H and C-A-S-H) and Ca(OH)_2_. These substances significantly improve the microstructure, as evidenced by a reduction in cracks and pores, along with the formation of hard crystalline structures, thereby enhancing the density and integrity of the structure, which ultimately leads to a significant increase in the strength of the stabilized soil. The experimental results demonstrate that LGGBS offers excellent mechanical properties and broad application potential for improving expansive soil.

## Figures and Tables

**Table 1:** Physical and Mechanical Properties of Expansive Soil.

Plastic Limit (%)	Liquid Limit (%)	Plasticity Index Ip (%)	Free Swell Ratio (%)	Montmorillonite Content (%)	Cation Exchange Capacity (mmol/kg)
28.75	45.49	16.74	51.0	33.61	19.8
**Maximum Dry Density (g/cm^3^)**		**Optimum Moisture Content (%)**	**Cohesion (kPa)**	**Internal Friction Angle (°)**	**Expansive Force at 95% Compaction** **(kPa)**
1.73		19.8	20.2	6.4	125.0

**Table 2:** The main components of expansive soil and slag.

Component	SiO_2_	Al_2_O_3_	CaO	MgO	SO_3_	Fe_2_O_3_
Expansive Soil	45.7	12.7	-	3.6	-	2.3
GGBS	34.2	17.6	34.0	19.8	1.62	1.01

**Table 3:** Pilot schemes.

Group	Alkaline Activator Content (%)	GGBS Content (%)	Modulus	Curing Duration (d)
1	15	-	1.0, 1.3, 1.5, 1.7, 2.0	7, 14, 28
2	5	-	1.5	
3	10	-		
4	15	-		
5	20	-		
6	-	10	-	
7	-	15	-	
8	-	20	-	
9	15	20	1.0, 1.3, 1.5, 1.7, 2.0	
10	5	10, 15, 20	1.5	
11	10	10, 15, 20		
12	15	10, 15, 20		
13	20	10, 15, 20		

## Data Availability

The original contributions presented in the study are included in the article, and further inquiries can be directed to the corresponding authors.
